# Shining light on multi‐drug resistant *Candida auris*: Ultraviolet‐C disinfection, wavelength sensitivity, and prevention of biofilm formation of an emerging yeast pathogen

**DOI:** 10.1002/mbo3.1261

**Published:** 2022-01-19

**Authors:** Richard M. Mariita, James H. Davis, Michelle M. Lottridge, Rajul V. Randive

**Affiliations:** ^1^ Product Engineering Department Crystal IS Inc., an Asahi Kasei Company Green Island New York USA

**Keywords:** antibiotic resistance, biofilm prevention, *Candida auris*, inactivation, UVC LED, yeasticidal

## Abstract

*Candida auris* is an emerging fungal superbug of worldwide interest. It is associated with high mortality rates and exhibits increased resistance to antifungals. Ultraviolet subtype C (UVC) light can be used to disinfect surfaces to mitigate its spread. The objectives of this study were (1) To investigate UVC disinfection performances and wavelength sensitivity of *C. auris*. (2) To evaluate the UVC dose required for the prevention of biofilm formation on stainless‐steel, plastic (polystyrene), and poly‐cotton fabric surfaces. *C. auris* was grown following standard procedures. The study utilized six different UVC LED arrays with wavelengths between 252 and 280 nm. Arrays were set at similar intensities, to obtain doses of 5–40 mJ cm^−2^ and similar irradiation time. Disinfection performance for each array was determined using log reduction value (LRV) and percentage reduction by comparing the controls against the irradiated treatments. Evaluation of the ability of 267 nm UVC LEDs to prevent *C. auris* biofilm formation was investigated using stainless‐steel, plastic coupons, and poly‐cotton fabric. Peak sensitivity to UVC disinfection was between 267 and 270 nm. With 20 mJ cm^−2^, the study obtained ≥LRV3. On stainless‐steel coupons, 30 mJ cm^−2^ was sufficient to prevent biofilm formation, while on plastic, this required 10 mJ cm^−2^. A dose of 60 mJ cm^−2^ reduced biofilms on poly‐cotton fabric significantly (*R* ^2^ = 0.9750, *p* = 0.0002). The study may allow for the design and implementation of disinfection systems.

## Graphical abstract

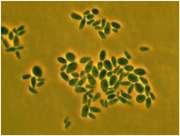

Disinfection performances of UVC LED arrays with wavelengths between 252 and 280 nm were investigated. Log reduction value (LRV) revealed that 267 and 270 nm arrays obtained the best disinfection performances, with 20 mJcm‐2 obtaining ≥LRV 3 in aqueous. Additionally, surface disinfection revealed that prevention of biofilm formation on stainless steel is achievable by dosing with 30 mJcm‐2, while on plastic 10 mJcm‐2 was enough for similar results. A dose of 60 mJcm‐2 significantly reduced biofilms on poly‐cotton fabric.

## 1 INTRODUCTION

*Candida auris* is a yeast that falls within the Metschnikowiaceae family (Chybowska et al., 2020), and is resistant to many commonly administered antifungal drugs (Kordalewska & Perlin, 2019; Lemons et al., 2019). It is currently a global emerging menace (Kordalewska & Perlin, 2019) that is considered to be an urgent threat by the Centers for Disease Control and Prevention (CDC) (Kadri, 2020) (Lone & Ahmad, 2019). Outbreaks have been documented in Europe (Ruiz‐Gaitán et al., 2018), Asia (Ahmad et al., 2020), South America (Nobrega de Almeida et al., 2021), and North America (Prestel et al., 2021). The CDC reports an in‐hospital mortality rate of up to 40% (Lemons et al., 2019), and the University of Maryland reports a mortality rate of 68% (Vila et al., 2020). Because of the high mortality rates, there is a need to not only have surveillance systems, such as those in New York (Zhu et al., 2020) but to also implement global mitigation strategies to help control the spread of *C. auris* (Ledwoch & Maillard, 2019). That is important because, unlike other *Candida* species, the deadly *C. auris*, which emerged simultaneously in three continents has had the unique ability that enabled it to spread globally, causing severe infections (Casadevall et al., 2019; Ku et al., 2018).

Of great consequence is that the emergence of *C. auris* may be a result of accelerating environmental and health trends (Chakrabarti & Sood, 2021). Although there has not been clear evidence that Covid‐19 patients are more susceptible to *C. auris* infections, higher rates in Covid‐19 treatment units such as the 39 cases have been reported in Florida by CDC during the pandemic (Prestel et al., 2021). Furthermore, the CDC has reported more outbreaks in Washington DC and Texas where 101 and 22 cases respectively were reported between January and April of 2021 (Lyman et al., 2021). The emergence and spread of *C. auris* have a possible association with climate change (Casadevall et al., 2019). If this is the case, developing mitigation strategies immediately may be necessary to prevent wide‐scale community outbreaks.

Of more concern is that *C. auris* displays several features that make mitigation and disinfection difficult. Some of these features include the ability to spread rapidly, persistence in the colonization of the skin and high‐touch surfaces (Horton Mark et al., 2020), and resistance to common disinfectants (Vila et al., 2020). *C. auris* is also resistant to conventional antibiotics such as fluconazole (Du et al., 2020; Zhu et al., 2020), and can survive for at least 28 days in the environment, all of which make it problematic in healthcare settings (Maslo et al., 2019). *C. auris* also causes bloodstream infections (Ruiz‐Gaitán et al., 2018). Its biofilms likely contribute to pathogenicity and aid in the spreading in healthcare settings due to the formation of surface‐adherent communities that can withstand desiccation and are resistant to antifungals (Horton & Nett, 2020). Specifically, *C. auris* biofilms are inherently resistant to polyenes (e.g., amphotericin B), azoles (e.g., fluconazole), and echinocandins (e.g., micafungin), the three main classes of antifungals (Sherry et al., 2017). Thus, finding effective and scalable non‐chemical disinfection strategies is essential.

Ultraviolet‐C (UVC) light has been demonstrated as effective by numerous studies (Cadnum et al., 2018; Chatterjee et al., 2020; Fu et al., 2020; Lemons et al., 2019; Maslo et al., 2019; Ponnachan et al., 2019). However, no UVC dose, wavelength, biofilm, or environmental conditions are often reported so it is not possible to compare results between studies or to use results to design or administer disinfection systems. Unfortunately, also because methylation and other produced chemicals can protect DNA from dimerization, it is difficult to determine the sensitivity of *C. auris* from theoretical models. Thus, the objectives of this study were to determine the wavelength sensitivity of this emerging pathogen, and the performance of UVC on preventing the formation of biofilms on stainless‐steel, plastic, and poly‐cotton fabric to allow for the design and implementation of disinfection systems for these high‐touch surfaces.

## 2 MATERIALS AND METHODS

### 2.1 *C. auris* strain and culture conditions

**Figure 1:**
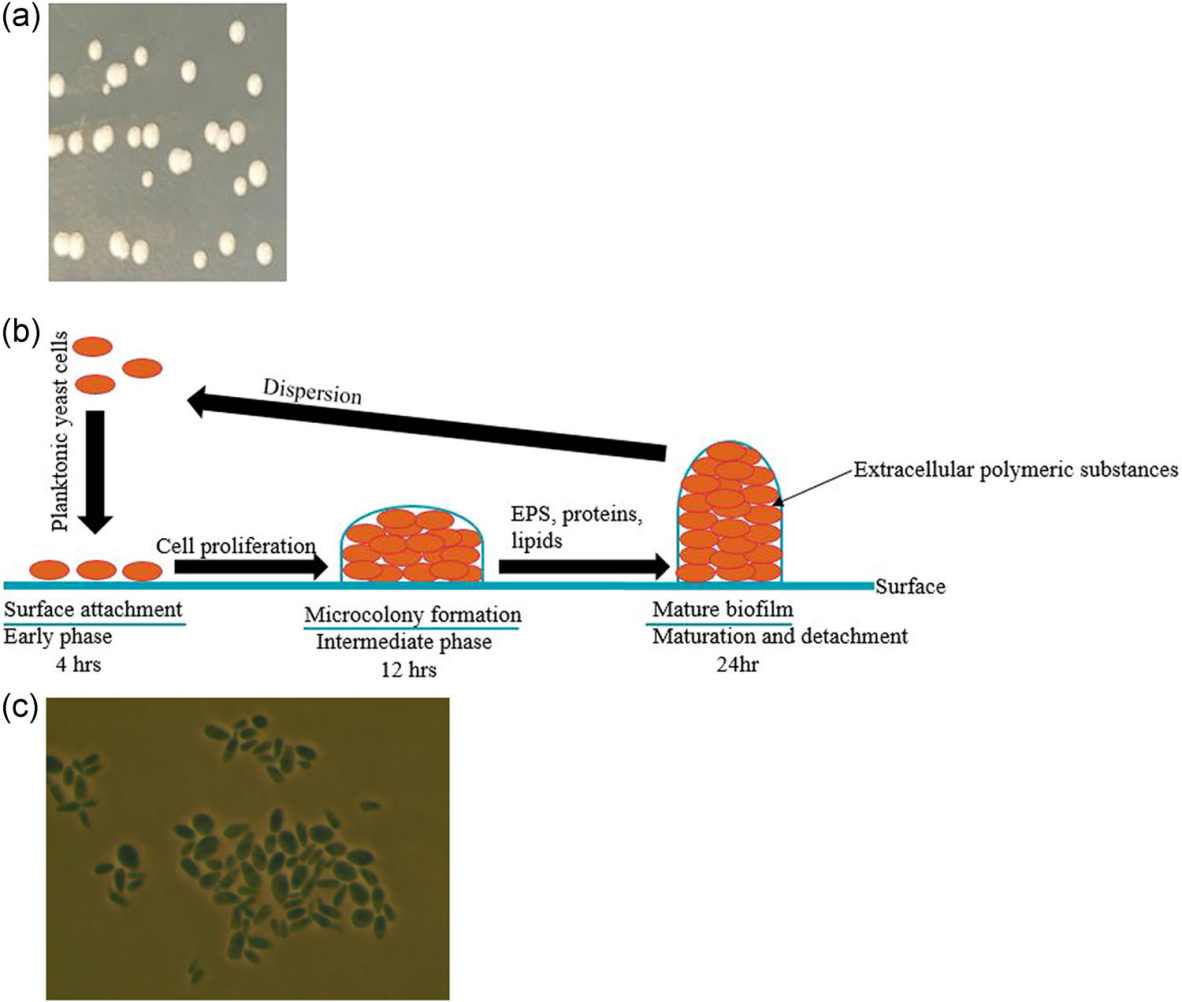
(a) Pure culture of *Candida auris* ATCC MYA‐5001 produced creamy white colonies with a smooth edge on YM agar after incubation at 37°C for 48 h; (b) *C. auris* forms robust biofilms after 24 h; (c) Images obtained on Zeiss Axiovert 200 Inverted Microscope at an objective magnification of ×100 confirmed ovoid yeast after Lactophenol Cotton Blue staining of 48 h old culture on YM agar

The strain used in this study, *C. auris* ATCC MYA‐5001 (ATCC strain designation B11220 = JCM 15448 = CBS 10913 = DSM 21092) was obtained from the American Type Culture Collection (ATCC). *C. auris* ATCC MYA‐5001 is 12,135,964 bp in size, with a %GC of 45.14%. It was propagated and incubated at 37°C for 48 h following the ATCC product sheet instructions. The strain was maintained in YM broth with 20% glycerol, and frozen at −80°C until use. ATCC Medium: 200 YM Medium (Agar or Broth) (pH 6.2) was used for the growth of pure cultures of *C. auris* (Figure 1a). The YM broth was also used to grow *C. auris* for biofilm formation (Figure 1b), as well as for growth towards the observation of *C. auris* for morphological confirmation and culture purity check via staining using Lactophenol Cotton Blue (Leck, 1999), and slide mounts observed on Zeiss Axiovert 200 Inverted Microscope (Figure 1c).

### 2.2 Disinfection stage set up and yeasticidal activity of UVC at different wavelengths

**Figure 2:**
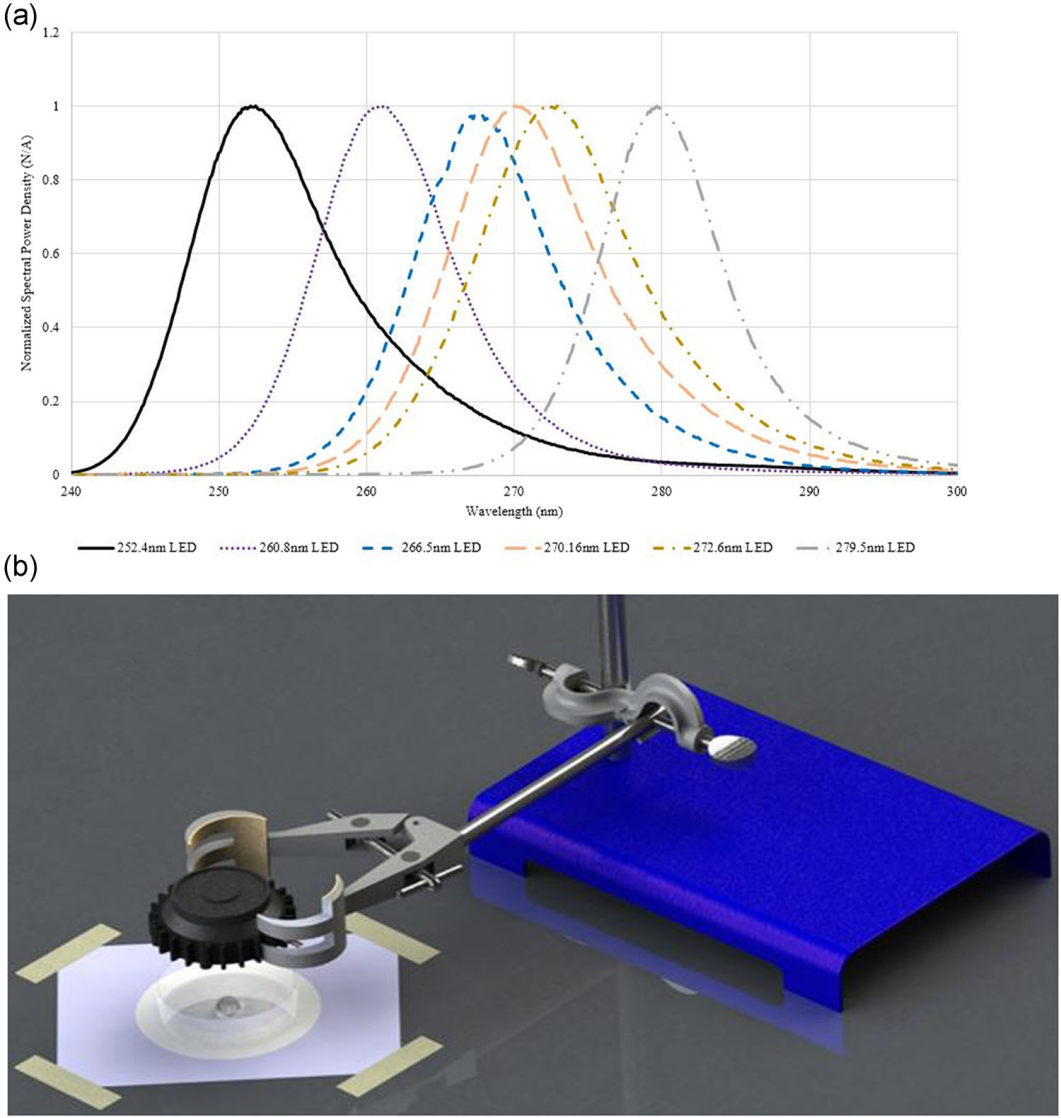
(a) Spectral analysis of arrays used in the study and (b) stage setup during the test

The peak wavelength of the different arrays used in the study ranged from 252.4 to 279.5 nm as confirmed by Maya Pro 2000 UVC spectrometer (Figure 2a). The irradiance over the 1 cm disinfection area was measured with an X1 MD‐37‐SC1‐4 optometer calibrated to 265 nm, with around 5% power variation between test wavelengths (Gigahertz, 2021).

The disinfection stage was set up using a ring stand that was placed above the disinfection zone. Inoculum for *C. auris* was prepared by growing the strain in YM broth at 37°C for 24 h. This was followed by centrifugation and resuspension of yeast cells in fresh 1× phosphate‐buffered saline (PBS) buffer (Cat # BP3994) (Fu et al., 2020). To standardize the resuspended culture, OD_600_ was adjusted using GENESYS 30 visible spectrophotometer (Thermo Scientific) to 0.15. This was to ensure the same concentration (8–9 × 10^5^ CFU/ml) of *C. auris* ATCC MYA‐5001 was obtained for each test. Additional confirmation to ensure approximately the same concentrations of test culture was performed by measuring the turbidity of the suspension to ensure an equivalent of 0.5 McFarland using a Sper Scientific 860040 Turbidity Meter (Sper Scientific) (Srivastava & Ahmad, 2020).

To investigate UVC yeasticidal activities for each array, 500 µl of *C. auris* with OD_600_ of 0.15 in sterile 1× PBS buffer was placed at the center of empty Petri dish and irradiated while in liquid phase at varying durations to obtain target constant doses of UVC for each array (Figure 2b). Then 500 µl of *C. auris* test culture for both irradiated and unirradiated (controls) were recovered using a pipette and spread plated on YM agar, and incubated at 37°C for 48 h. As in a previous study (Mariita & Randive, 2021), colonies were then counted for use in the calculation of logarithmic reduction values (LRV) and % reduction by comparing the controls (not irradiated with UVC) against the UVC irradiated treatments. Experiments were performed in triplicate.

### 2.3 Evaluation of UVC for prevention of biofilm formation against *C. auris*

Fresh *C. auris* cultures were made from freezer stocks by inoculating the YM broth and growing aerobically overnight at 37°C in baffled flasks in a shaker running at 180 rpm for 24 h. Overnight cultures were centrifuged once at 4500 rpm for 10 min to remove media and suspended in 1× PBS buffer before adjusting optical density at 600 (OD_600_) to 0.15. Stainless‐steel, plastic (polystyrene), and poly‐cotton fabric coupons were then inoculated with 200 μl of the suspension. To allow for adherence of *C. auris* to the coupon surfaces, the inoculated materials were incubated for 1.5 h at 37°C while shaking at 100 rpm (Khan et al., 2012). Following the adhesion phase, the coupons were washed twice using 300 µl of 1× PBS buffer to remove loosely attached cells. The 267 nm LED array was driven at 500 mA and 6.43 V with a UVC intensity of 1.00 mW cm^−2^ confirmed using the optometer. To determine UVC dose that offers significant or total prevention of biofilm formation, UVC was applied for 5, 10, 30, and 40 s, to yield UVC doses of 5, 10, 30, and 40 mJ cm^−2^ respectively on the contaminated surfaces of stainless‐steel and plastic coupons. For poly‐cotton fabric, 30, 40, and 60 mJ cm^−2^ were used as target doses. After irradiation, a total of 100 µl freshly made 1% YM broth, was added to all treatments. Experiments were done in triplicates. Coupons were incubated at 37°C for 24 h at 90% relative humidity (RH) (Sanchez et al., 2016). Incubator RH adjustments were achieved using 35.89 g of NaCl (Fisher Scientific S271‐500) in 100 ml of deionized water in a beaker. Onset HOBO loggers were placed in the incubation chambers to monitor and confirm RH conditions during the incubation period.

#### 2.3.1 Biofilm analysis using crystal violet assay

After 24 h the biofilms were harvested for analysis following a crystal violet assay modified protocol from Sherry et al. (2017) and Gulati et al. (2018). Specifically, coupons were processed by first rinsing them three times using 300 μl 1× PBS buffer, then air drying for 45 min. They were then stained using 100 µl of 1% crystal violet (Signa cat# V5265‐500) for another 45 min. This was followed by washing them four times with 300 μl sterile water to remove excess stain. Destaining was performed using 1000 μl 30% glacial acetic acid solution (Merck Millipore Cat #1.59166.0500). After destaining, 1000 μl of the 30% glacial acetic acid (destaining solution) was used for OD measurement at 595 nm.

#### 2.3.2 Biofilm analysis using CFU/ml assay

After 24 h of biofilm growth, the coupons were rinsed three times in 300 μl 1× PBS buffer to rid of any planktonic bacteria. This was followed by the addition of 1 ml 1× PBS buffer for sonication. The sonicator (Branson Model # CPX 2800) was first degassed for 5 min, before sonication for 20 min, followed by vortexing to mix. Recovery was done by growing on YM agar. Incubation done at 37°C at 90% humidity for 48 h and colony counts used in statistical analysis.

### 2.4 Optical microscopy

The surface properties of stainless‐steel, plastic, and poly‐cotton fabric coupons were investigated using an Olympus (BX41M‐LED) microscope. Exposure time was set at 35, 60, and 175 ms respectively.

### 2.5 Statistical analysis

To trace the trend related to disinfection performance for each array, linear regression analysis (simple exponential model) was carried out. Additionally, an unpaired *t*‐test (two‐tailed) was used to measure the statistical significance of the impact of UVC in the prevention of biofilm formation. Statistical analysis was performed using GraphPad Prism 9.1.2 (GraphPad Software, Inc.).

## 3 RESULTS

### 3.1 Wavelength sensitivity of *C. auris*

**Figure 3:**
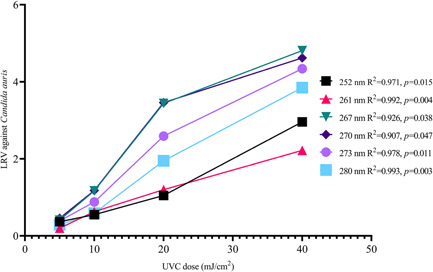
Disinfection performance against *Candida auris* versus UVC dose obtained from different arrays. Similar UVC doses for each array were used during testing. Performance of arrays increases with dose. All arrays were designed to deliver the same amount of dose for a similar amount of time to avoid bias. UVC LEDs emitting radiation of 260–270 nm obtained higher disinfection with less time. LED, light‐emitting diode; UVC, ultraviolet‐C

The results of the wavelength sensitivity tests revealed that 267–270 nm peak wavelengths offered higher disinfection performance (Table 1 and Figure 3). The 267 and 270 nm have a similar effect, with the fastest inactivation rate of an average of 0.13 LRV/mJ^−1^ cm^2^. Disinfection efficiency declined after a 3.5 log reduction for both. The 252 and 261 nm wavelengths performed the worst. Linear regression analysis and trendlines revealed a significant association between all arrays and their disinfection efficacy at 5, 10, 20, and 40 mJ cm^−2^ (Figure 3), while emphasizing the effectiveness of UVC emission wavelengths of 267–270 nm.

**Table 1:** UVC efficacy in inactivating *Candida auris* revealed that with 267 and 270 nm peak wavelengths, LRV 3 (99.9% reduction) is obtained

Peak wavelength (nm)	Time (s)	Dose (mJ cm^−2^)	Controls (CFU ml^−1^)	UVC dosed (CFU ml^−1^)	LRV	% reduction	Susceptibility constant (k) (cm^2^ mJ)
252	5	5	8.60E + 05	3.67E + 05	0.37	57.326	0.0691
	10	10	8.60E + 05	2.43E + 05	0.55	71.744	
	20	20	8.60E + 05	7.67E + 04	1.05	91.081	
	40	40	8.60E + 05	9.33E + 02	2.96	99.892	
261	5	5	8.63E + 05	5.47E + 05	0.20	36.617	0.0565
	10	10	8.63E + 05	2.03E + 05	0.63	76.477	
	20	20	8.63E + 05	5.50E + 04	1.20	93.627	
	40	40	8.63E + 05	5.21E + 03	2.22	99.396	
267	5	5	6.40E + 05	2.50E + 05	0.41	60.938	0.1294
	10	10	6.40E + 05	4.33E + 04	1.17	93.234	
	20	20	6.40E + 05	2.33E + 02	3.44	99.964	
	40	40	6.40E + 05	1.00E + 01	4.81	99.998	
270	5	5	9.53E + 05	3.33E + 05	0.46	65.058	0.126
	10	10	9.53E + 05	6.33E + 04	1.18	93.358	
	20	20	9.53E + 05	3.33E + 02	3.46	99.965	
	40	40	9.53E + 05	2.33E + 01	4.61	99.998	
273	5	5	8.00E + 05	3.27E + 05	0.39	59.125	0.111
	10	10	8.00E + 05	1.07E + 05	0.88	86.625	
	20	20	8.00E + 05	2.03E + 03	2.59	99.746	
	40	40	8.00E + 05	3.67E + 01	4.34	99.995	
280	5	5	4.07E + 05	2.07E + 05	0.29	49.140	0.0889
	10	10	4.07E + 05	1.70E + 05	0.38	58.537	
	20	20	4.07E + 05	2.87E + 04	1.16	93.000	
	40	40	4.07E + 05	4.00E + 01	4.01	99.990	

*Note*: The UVC inactivation of *C. auris* was carried out in an aqueous solution.

### 3.2 Biofilm prevention activity

**Figure 4:**
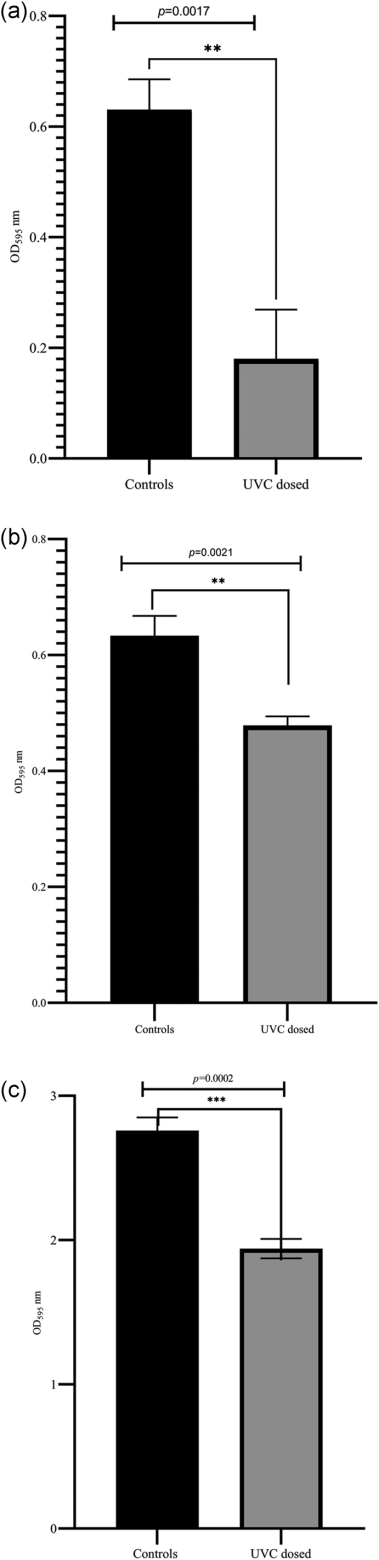
A bar graph showing the performance of 267 nm UVC LED array against biofilm formation activity. (a) UVC dose of 10 mJ cm^−2^, when applied at the early phase of biofilm formation, significantly (*R* ^2^ = 0.9331, *p*  =  0.0017) inhibited biofilm formation on stainless‐steel surfaces, while 30 mJ cm^−2^ completely inhibited biofilm formation. (b) On plastic, when 5 mJ cm^−2^ was applied, there was significant prevention of biofilm formation (*R* ^2^ = 0.9265, *p*  =  0.0021), while 10 mJ cm^−2^ prevented biofilm formation. (c) On poly‐cotton fabric, 60 mJ cm^−2^ had a significant impact on the prevention of biofilm formation (*R* ^2^ = 0. 0.9750, *p*  =  0.0002). Error bars show a 95% confidence interval (CI). **Statistically significant difference. Experiments were done in triplicates. LED, light‐emitting diode; UVC, ultraviolet‐C

**Figure 5:**
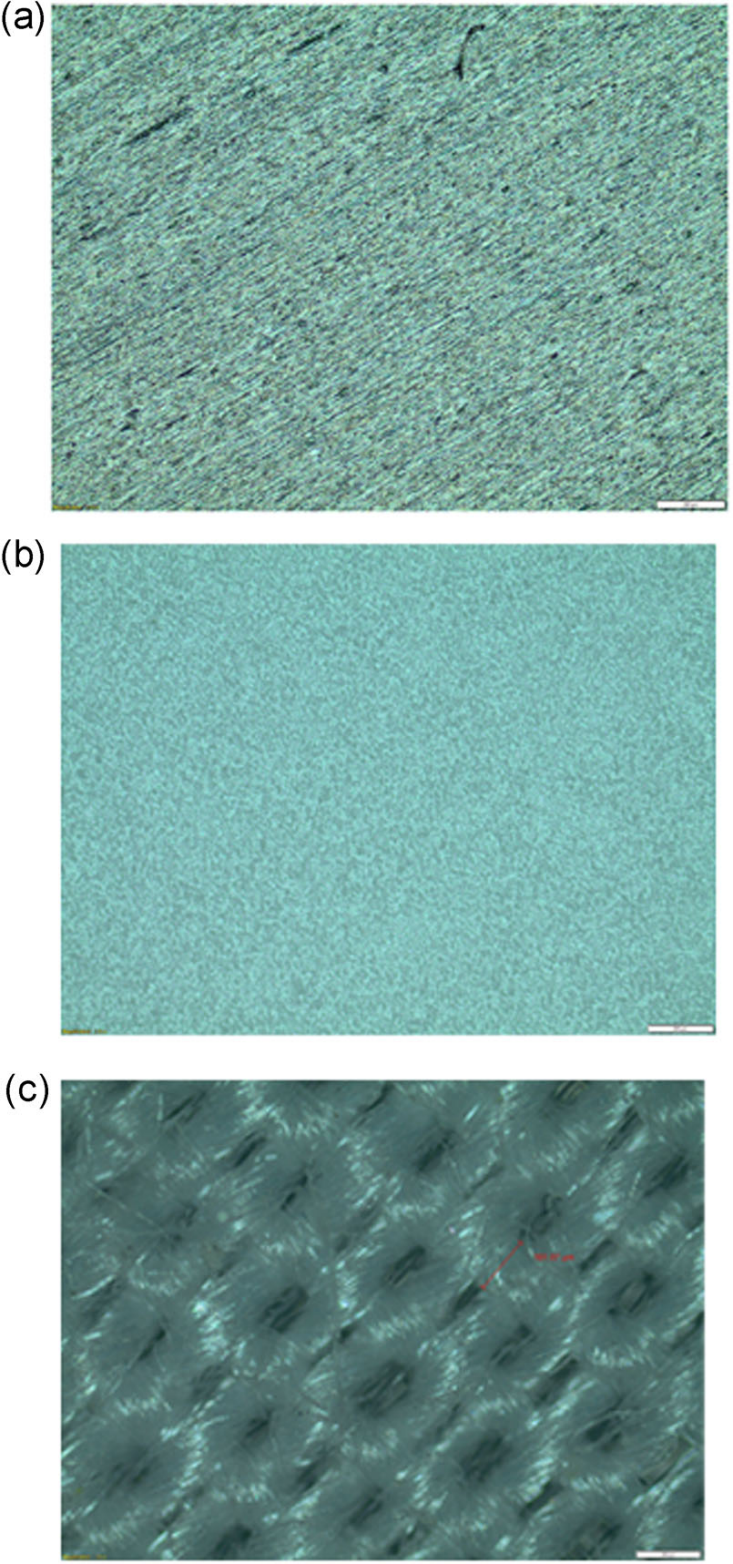
Microstructural differences between (a) stainless‐steel, (b) plastic, and (c) poly‐cotton fabric coupons used in the biofilm prevention analysis. Biofilm prevention at 267 nm was obtained with 30 and 10 mJ cm^−2^ on stainless‐steel and plastic coupons, respectively. A dose of 60 mJ cm^−2^ reduced biofilms on poly‐cotton fabric significantly (*R* ^2^ = 0.9750, *p* = 0.0002), although was not enough to completely prevent biofilm formation. Material differences could explain observations

At 267 nm, UVC LEDs exhibited antibiofilm activity. A dose of 10 mJ cm^−2^, when applied at the early phase of biofilm formation, significantly (*R* ^2^ = 0.9331, *p*  =  0.0017) inhibited biofilm formation on stainless steel surfaces (Figures 4a and 5a), while completely inhibiting biofilm formation with 30 mJ cm^−2^. On plastic, it was possible to prevent biofilm formation with 1 mW cm^−2^ via static dosing at 10 mJ cm^−2^ (irradiation 10 s) (Figure 5b). Against poly‐cotton fiber, 60 mJ cm^−2^ significantly reduced biofilm formation (*R* ^2^ = 0.9889, *p* ≤ 0.0001) (Figure 5c), but was not enough to prevent biofilm formation. These observations could be explained by the surface microstructure differences in the test surfaces (Figure 5).

## 4 DISCUSSION

### 4.1 UVC dose requirements for planktonic cultures and biofilm prevention

*C. auris* is less susceptible to UVC than previously studied bacterial pathogens (MRSA, VRE, and *Acinetobacter baumannii*) associated with HAIs (Mariita & Randive, 2021). This is likely because *C. auris*, which is a yeast (unicellular eukaryote), is a higher organism, with seven chromosomes (Muñoz et al., 2021), as opposed to the bacterial strains which mainly have one chromosome (Harrison et al., 2010). Overall, these results show that *C. auris* ATCC MYA‐5001 is most sensitive to 267–270 nm light and that it requires 20 mJ cm^−2^ to achieve a 3‐log reduction at 267 nm in aqueous. Irradiation at 267 nm with 30 and 10 mJ cm^−2^ was sufficient to prevent biofilm growth on stainless‐steel and plastic coupons, respectively. The significant reduction of biofilm formation on poly‐cotton fabric by the study confirms how critical it is for design systems to verify performance against target surfaces. This is because one of the limitations of UVC, like many other disinfectants, is that one solution cannot be applied across the board.

### 4.2 Regulatory and disinfection considerations

The International Sanitary Supply Association (ISSA) has established cleaning times based on a square foot driven estimate (Insero, 2019). Among other considerations is the recommendation that nursing and healthcare environmental services (EVS) personnel doing the cleaning should wait until sufficient time has elapsed to allow for enough air changes to dilute or remove potentially infectious particles (CDC, 2019). UVC products, if properly employed, can be used during the recommended wait time, thus saving additional time, and preventing the spread of pathogens by supplementing existing mechanical ventilation (Kahn & Mariita, 2021). Additionally, one of the best EPA registered *C. auris* disinfection products is dodecylbenzenesulfonic acid (Ecolab Inc., EPA reg. number: 1677‐262) (US EPA, 2020), which needs 60 s of contact time to effectively disinfect hard nonporous surfaces. The UVC arrays under this study, without any optimization, take <40 s to obtain >99.99% microbial reduction (>LRV 4), with no chemical residues left behind (Figure 2a,b). Exposure time and distance are important in the inactivation of *C. auris* (de Groot et al., 2019), but even more critical is the amount of UVC dosage and inactivation efficacy that can be obtained for each exposure time and at a given distance.

Previous findings have revealed sources of variability in the yeasticidal effect of UVC. One source of variability could be the overlapping of the microbes during disinfection, which could reduce UVC penetrability leading to lower log reduction than expected in real applications (Cevenini et al., 2020). Such overlapping is due to the use of a high concentration of microorganisms during laboratory experiments. Although the sensitivity of the microbial population is not uniform, and thus LRV is not strictly linear with dose, the results from this study imply the prediction from the model by Lemons et al. (2019), which projected that LRV5 (99.999% reduction) can be obtained with 66–110 mJ cm^2^ of UVC dose, is likely.

### 4.3 Effects of test strains and test conditions

It should be emphasized that this study only used one drug‐resistant strain from clade II, *C. auris* type strain (ATCC MYA‐5001 = B11220 = JCM 15448 = CBS 10913 = DSM 21092) isolated from the human auditory canal at Tokyo Metropolitan Geriatric Hospital (Satoh et al., 2009). Different strains (for instance, those from clades I, III, IV, and V) may yield slightly different results due to possible differences in sensitivities. Additionally, although temperature and humidity are reported, the effects of temperature and humidity were not explored as the purpose of this study was not to investigate the impact of temperature and humidity on disinfection efficacy. Non‐linearities between exposure and disinfection, although known and observed, were not explored in‐depth. Also, due to the inaccessibility of some LED packages in wavelengths in the whole study target range, the 252 and 261 nm LEDs were in a package with a ball lens, perhaps explaining their performance. In addition to those factors, light uniformity, power output variability, and system lifetime can be variable between designs and should be considered during design and testing.

## 5 CONCLUSIONS

With controlled UVC irradiance of 1 mW cm^−2^, the study obtained a 99.998% reduction with 40 mJ cm^−2^ with 267 and 270 nm arrays after 40 s. Additional studies are needed in a real hospital environment to assess the real‐life applications and determine the cumulative impact of repeated disinfection sessions. Further, the use of UVC radiation is feasible for the disinfection of *C. auris* on permanent surfaces like plastics and metals and may constitute a valuable adjunct to routine cleaning in healthcare environments. The design and implementation of germicidal UVC‐LED devices could minimize the use of corrosive and irritating chemicals, and reduce sanitization wait time because wet decontamination requires wait time for effectiveness (Richard et al., 2018). Additionally, this study is important as it adds to the knowledge in determining effective infection control by testing typical materials and fabrics commonly found in healthcare settings. Because UVC dose, wavelength, and environmental conditions are critical parameters that determine disinfection efficacy, this knowledge will help designers and researchers to efficiently come up with UVC LED‐based solutions that can contribute towards infection control.

## CONFLICT OF INTERESTS

Richard Mariita, James Davis, Michelle Lottridge, and Rajul Randive all receive salaries from Crystal IS, an Asahi Kasei company that manufactures UVC‐LEDs.

## ETHICS STATEMENT

None required.

## AUTHOR CONTRIBUTIONS

**Richard Mariita**: Conceptualization (Lead), Formal analysis (Lead), Investigation (Lead), Methodology (Lead), Supervision (Lead), Validation (Lead), Writing—original draft (Lead), Writing—review & editing (Lead). **James Davis**: Formal analysis (Supporting), Investigation (Supporting), Methodology (Supporting), Validation (Supporting), Visualization (Equal), Writing—original draft (Supporting), Writing—review & editing (Supporting). **Michelle Lottridge**: Investigation (Supporting), Writing—review & editing (Supporting). **Rajul Randive**: Formal analysis (Supporting), Validation (Supporting), Writing—review & editing (Supporting).

## Data Availability

All data generated or analyzed during this study are included in this published article. Spectral analysis raw data can be accessed in figshare via https://doi.org/10.6084/m9.figshare.16776112.v3. Inactivation rate constant (k) analyses can be accessed in figshare via https://doi.org/10.6084/m9.figshare.16989481.v1.
